# Spatial Distribution Characteristics and Driving Factors of Formicidae in Small Watersheds of Loess Hilly Regions

**DOI:** 10.3390/insects16060630

**Published:** 2025-06-15

**Authors:** Yu Tian, Fangfang Qiang, Guangquan Liu, Changhai Liu, Ning Ai

**Affiliations:** 1 Key Laboratory of Applied Ecology on the Loess Plateau of Shaanxi Higher Education Institutions, College of Life Sciences, Yan’an University, Yan’an 716000, China; 18084932271@163.com (Y.T.); ffqiang@yau.edu.cn (F.Q.); 2 China Institute of Water Resources and Hydropower Research, Beijing 100038, China; gqliu@iwhr.com

**Keywords:** loess hilly area, Formicidae, spatial distribution, geographically weighted regression model (GWR)

## Abstract

This study takes the Jinfoping Small Watershed in the Loess Hilly Region as the research area. Through field investigation and laboratory analysis, combined with methods such as spatial autocorrelation analysis, the ordinary least squares method (OLS), and the geographically weighted regression model (GWR), it deeply explores the spatial distribution characteristics and driving factors of Formicidae in the study area. The research results are as follows: (1) Spatial autocorrelation analysis indicates that the distribution of Formicidae is significantly regulated by spatial dependence and has significant spatial autocorrelation (global Moran’s I = 0.332; *p* < 0.01). (2) The spatial visualization analysis of the GWR model reveals that soil physical and chemical properties and topographic factors have local influences on the spatial distribution of Formicidae. Available phosphorus (AP) and slope (SLP) were significantly positively correlated with the number of ants. Hydrogen peroxidase (HP) and topographic relief (TR) were significantly negatively correlated with the number of ants. This study reveals the spatial distribution pattern of Formicidae in the Loess Hilly Region and its complex relationship with environmental factors, and clarifies the importance of considering spatial heterogeneity when analyzing ecosystem processes. The research results provide a scientific basis for the protection and management of soil ecosystems, and also offer new methods and ideas for future related research.

## Simple Summary

This study investigates the spatial distribution patterns of Formicidae (ants) and their driving factors in the Jinfoping Small Watershed, located in the Loess Hilly Region of China. Using field surveys, lab analysis, and spatial statistical methods—including spatial autocorrelation analysis, ordinary least squares (OLS), and geographically weighted regression (GWR)—we found that ant distribution showed significant spatial clustering (Moran’s I = 0.332; *p* < 0.01). Key environmental factors influencing Formicidae populations include the available phosphorus (AP) and slope (SLP), which have positive effects, while hydrogen peroxidase (HP) and topographic relief (TR) negatively affect Formicidae abundance. The results highlight the importance of spatial heterogeneity in understanding ecosystem processes and provide valuable insights for soil ecosystem conservation. This study offers a scientific foundation for ecological management and suggests new approaches for future research on soil biodiversity.

## 1. Introduction

As the Loess Hilly Area is one of the regions with the most fragile ecological environment in China, the stability and sustainability of its ecosystem have always attracted much attention [1]. Over the past few decades, with the implementation of large-scale ecological restoration projects, the vegetation coverage in this area has significantly increased, and the ecological environment has been improved to a certain extent [2,3]. However, the restoration of an ecosystem is a complex process involving interactions among multiple biotic and abiotic components. Formicidae serve as pivotal functional groups within soil fauna communities [4], functioning as “ecosystem engineers” [5] that play an indispensable role in maintaining soil health and ecosystem stability through regulating ecological processes such as organic matter decomposition, soil structure improvement, and nutrient cycling [6]. The distribution of ant fauna is influenced by multiple interacting factors, including soil physicochemical properties, topographic features, and vegetation types [7]. The Loess Hilly Region, characterized by a complex topography and diverse vegetation, provides a natural laboratory for investigating ant–environment relationships. Existing studies have demonstrated pronounced spatial heterogeneity in ant assemblages across different ecosystems [8].

Currently, research on ants in the Loess Hilly Region has primarily focused on species composition and ecological functions [9,10,11,12], while investigations into spatial distribution patterns and their underlying drivers remain limited. Most existing studies employ conventional quadrat surveys and descriptive statistical approaches [13,14], which are insufficient for comprehensively elucidating fine-scale heterogeneity in ant nest distribution. Although advanced spatial analysis techniques, such as spatial point pattern analysis and geostatistics, have been successfully applied in ant ecology research [15], their implementation in studying ant spatial distribution in the Loess Hilly Region is still in its nascent stage. The application of spatial analysis methods such as geographically weighted regression (GWR) in ecological research has gradually increased [16] but they have not yet been widely used in the study in the Loess Hilly Area. This study takes the Jinfoping Small Watershed in the Loess Hilly Region as the research area. Through systematic field investigation and laboratory analysis, combined with methods such as spatial autocorrelation analysis, the ordinary least squares method (OLS), and the geographic weighted regression model (GWR), it deeply explores the spatial distribution characteristics of Formicidae, as well as the relationship between their influencing factors. The innovation of this study lies in the following: (1) For the first time, multiple spatial analysis methods are comprehensively applied in the Loess Hilly Area to fully reveal the spatial distribution pattern of Formicidae and the influencing factors. (2) Through the GWR model, this study conducts an in-depth analysis of how soil chemical properties and topographic factors influence the spatial distribution patterns of Formicidae at local scales, providing a new perspective for clarifying the complex relationship between Formicidae and environmental factors. (3) The research results will provide a scientific basis for the evaluation of biodiversity conservation and ecological restoration benefits in the Loess Hilly Region’s ecosystem, promoting the comprehensive sustainable development and scientific assessment of the regional ecological environment.

## 2. Materials and Methods

### 2.1. Overview of the Research Area

The study area is located in the Jinfoping Small Watershed of the Loess Hilly Region (108°23′09″−108°23′20″ E, 37°21′09″−37°21′20″ N), with a total area of 41 square kilometers. The landform is a loess hilly and gully area, with an altitude of 1500–1600 m. The overall terrain is higher in the west and lower in the east.

The climate of the study area belongs to the temperate continental monsoon climate. The annual average rainfall is 483.7 mm, unevenly distributed, mainly concentrated from July to September. The annual average temperature is 7.8 °C, and the annual average sunshine duration is 2400 h. The soil type is yellow soft soil, with a texture of light sandy loam, which is loose and porous. It has poor fertilizer and water retention, and is highly prone to erosion. Erosion is more intense on steep slopes. The content of organic matter nitrogen and phosphorus nutrients is low. The main vegetation types include *Hippohgae rhamnoides*, *Populus simonii*, *Prunus armeniaca var. ansu*, *Prunus davidiana*, *Pinus tabuliformis*, *Robinia pseudoacacia*, *Populus hopeiensis*, *Potentilla chinensis*, *Lespedeza Mainly daurica*, and *Thymus mongolicus*.

### 2.2. Study Plot Establishment

**Figure 1:**
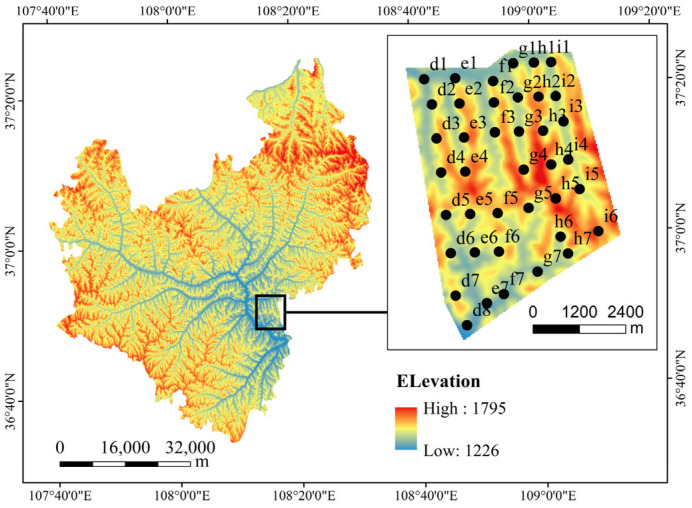
Elevation and sampling plot distribution map of Wuqi County.

Based on comprehensive field surveys and investigations of the Jinfoping Small Watershed, our research team employed a uniform grid-based sampling method for plot establishment according to the study area’s size. Considering the actual topography and vegetation distribution patterns, the grid spacing was set between 700 and 800 m. During field sampling, we strategically selected sampling plots to ensure both representativeness and spatial uniformity, with each plot carefully chosen to reflect the watershed’s natural characteristics. The sampling plots were primarily located in areas featuring typical vegetation types (arbors, shrubs, and grasslands). A total of 40 sampling plots were established during the study period. Based on the actual distribution of the sampling sites, we created a sampling plot distribution map (see Figure 1, elevation and sampling plot distribution map of Wuqi County).

### 2.3. Collection and Identification of Soil Fauna

Collection of soil animals: Within each sampling point, three 5 m × 5 m subplots were established in a triangular arrangement (a triangle-shaped pattern) as replicates for each vegetation type present. Each subplot contained three sampling quadrats. Macro-soil animals: Collected by hand-picking method from different soil layers (litter layer, 0–5 cm, 5–10 cm, 10–15 cm; quadrat size, 25 cm × 25 cm; depth, 15 cm). Specimens were preserved in 75% alcohol bottles, labeled, and transported to the laboratory for microscopic identification and classification. Medium and small soil animals: Soil samples from different layers (litter layer, 0–5 cm, 5–10 cm, 10–15 cm; quadrat size, 10 cm × 10 cm; depth, 15 cm) were collected using a 5 cm diameter and 5 cm height cutting ring. Samples were placed in sealed plastic bags and brought to the laboratory. Specimens were extracted using Tullgren funnels (SOIL-F2021, Watkins& Doncaster, UK) with 60 W incandescent lamp illumination for 48 h, and then preserved in 75% alcohol.

Soil animal identification: The collected soil animals were identified according to the large category method proposed by Junichi Aoki (1973). Stereoscopic microscopy (Nikon SMZ1270, Nikon Corporation, Tokyo, Japan) was used for identification, and the bibliography is mainly bibliography of Chinese Soil Fauna [17] and Chinese Subtropical Soil Fauna [18]. The number of individuals and groups of soil animals in each quadrat were counted. Since soil animals have different functions at different stages of development, adults and larvae are counted separately, and most adults are identified as families, while larvae or nymphs are identified as orders (suborders).

### 2.4. Soil Parameter Analysis

In this study, a systematic stratified sampling method was employed across 40 soil fauna survey sites. At each site, three soil profiles were arranged in a triangular pattern. From each profile, soil samples were collected at four depth intervals (0–10 cm, 10–20 cm, 20–30 cm, and 30–40 cm), with three replicate subsamples taken per depth, and then homogenized to form one representative composite sample. This sampling protocol yielded a total of 480 composite soil samples (40 sites × 3 profiles × 4 depth intervals) for subsequent analysis. For the determination of soil physical and chemical indices, refer to Soil Physical and Chemical Analysis [19] and Soil Agrochemical Analysis [20]. The names, measurement methods, and specific values of soil indicators are shown in Table 1.

### 2.5. Terrain Factor Data Sources

Based on the DEM data of Jinfoping Basin, the Spatial Analyst module of Arcgis10.8 was used to extract 7 topographic indices, including elevation, for analysis and calculation. The names, acquisition methods, and specific values of terrain indicators are shown in Table 2.

### 2.6. Research Method

#### 2.6.1. Global Spatial Autocorrelation

The global Moran’s I was calculated using ArcGIS (10.8.1) software to determine whether Formicidae exhibited spatial autocorrelation. The calculation formula is as follows (Equation (1)):

(1) $$I=\frac{n\sum_{i=1}^{n}\sum_{j=1}^{n}W_{ij}\left(x_{i}-x\right)\left(x_{j}-x\right)}{\sum_{i=1}^{n}\sum_{j=1}^{n}W_{ij}\sum_{i=1}^{n}{\left(x_{i}-\bar{x}\right)}^{2}}$$

In Formula (1), n is the number of spatial samples, $x_{i}$ and $x_{j}$ correspond to the attribute values of points i and j in the space, respectively, $W_{ij}$ corresponds to the spatial weight matrix, and $\bar{x}$ is obtained by averaging $x_{i}$ and $x_{j}$. I is the global Moran’s I index, and the value range of I is [−1, 1]. I > 0 indicates that there is a positive spatial correlation in the study area, I < 0 indicates that there is a negative spatial correlation in the study area, and I = 0 indicates that there is no spatial correlation in the study area [21]. The exponential value of Moran’s I is obtained by calculating the z-score and the *p*-value. The z-score is a standard deviation multiple that reflects the dispersion of the data set [22]. The *p*-value indicates the probability, and when *p* is small, it means that the observed spatial pattern is unlikely to arise from a random process (small probability event), and thus the null hypothesis can be rejected. The confidence levels corresponding to the z-scores and *p* values are shown in Table 3 below [23].

#### 2.6.2. OLS and GWR Models

The ordinary least squares (OLS) model is a commonly used linear regression analysis method in statistical analysis [25,26,27,28]. It is used to describe the relationship between independent variables (independent variables) and dependent variables (dependent variables) [29]. The basic form of the OLS model is shown as follows in Formula (2):

(2) $$y_{i}=\beta_{0}+\sum_{i=1}\beta_{k}x_{ik}+\varepsilon_{i}$$

In Equation (2), y_i_ is the dependent variable of the ith sample point (the distribution number of Formicidae at each point), X_i__k_ is the kth independent variable of the ith sample point (the influential factor affecting the distribution of Formicidae), β_o_ is the intercept (constant term) of the linear regression equation, β_k_ is the regression coefficient of the kth independent variable, and ε_i_ is the random error.

Geographically weighted regression (GWR) is a local regression method that considers spatial changes, enabling it to better capture spatial heterogeneity and local relationships [30,31]. In this paper, the GWR model is used to further explore the factors affecting the spatial distribution of Formicidae. The calculation formula is as follows:

(3) $$Y_{i}=\beta_{0}\left(u_{i},v_{i}\right)+\sum_{k=1}^{n}\beta_{k}\left(u_{i},v_{i}\right)X_{ik}+\varepsilon_{i}$$

In Formula (3), i=1,2,……n. (u_i_,v_i_) is the spatial latitude and longitude coordinate point of the i th sample point; $\sum_{k=1}^{n}\beta_{k}\left(u_{i},v_{i}\right)$ is the regression coefficient of the variable X_ik_; X_ik_ is the influencing factor; ε_i_ is the random error term, ε_i_ ~ N(0,δ^2^); n is the sample size. In view of the relatively small sample size and the principle of optimal bandwidth selection, the AICc method is adopted in this paper.

In this paper, the following two models are used: the geographically weighted regression (GWR) model and the ordinary least squares (OLS) model. In order to verify the validity and accuracy of these two models, the following three key evaluation indicators were used: the goodness-of-fit R2, adjusted R2, and AICc (Akaike Information Criterion) value. When the value of the AICc is the smallest, the fitting effect of the model is the best.

#### 2.6.3. Variance Inflation Factor (VIF)

The primary purpose is to verify the presence of multicollinearity. A VIF value greater than 7.5 indicates that the variable may have multicollinearity [32]. The calculation formula is as follows:

(4) $${VIF}_{j}=\frac{1}{1-R_{j}^{2}}$$

In Equation (4), R_j_ denotes the multiple correlation coefficient for the ith variable X_j_ (i = 1, 2… k; i ≠ j); $R_{j}^{2}$ represents the coefficient of determination obtained by regressing X_j_ (as the dependent variable) against the other variables X_j_ (i = 1, 2… k; i ≠ j) [33]. After applying the OLS model, if any VIF value exceeds 7.5, the corresponding variable should be excluded, and the OLS regression analysis should be reconducted.

#### 2.6.4. Radial Basis Function (RBF) Interpolation Method

As a spatial interpolation method [34], the Radial Basis Function (RBF) approach primarily employs basis functions to determine optimal weights from surrounding known data points to interpolated grid nodes. The resulting interpolated surface must pass through every known sample point within the study area. This method belongs to a category of artificial neural network approaches [35]. The core advantages of RBFs lie in ensuring the interpolated surface precisely passes through all input points, making it suitable for high-precision scenarios; flexibly adapting to spatial variations at different scales by adjusting the basis function parameters (e.g., the bandwidth of Gaussian kernels); and maintaining robust interpolation performance for discrete and irregularly distributed data points [36,37,38]. In this study, the Radial Basis Function interpolation method was employed to spatially visualize the distribution of ant species abundance and the regression coefficients of the geographically weighted regression (GWR) model. This approach provides a more intuitive representation of the spatial distribution patterns of ant populations and their spatially non-stationary relationships with environmental factors.

## 3. Results and Analysis

### 3.1. Spatial Distribution and Spatial Autocorrelation Analysis of Formicidae

**Figure 2:**
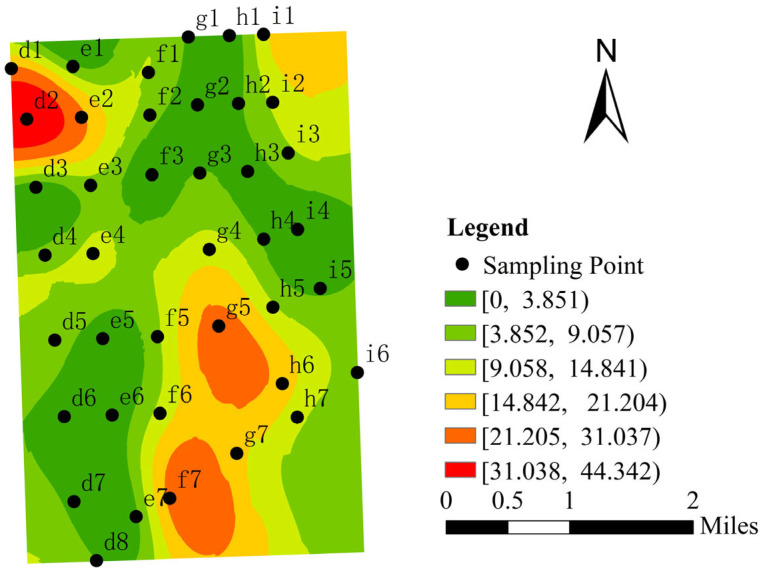
Spatial distribution map of Formicidae.

Spatial analysis of Formicidae distribution patterns was performed using Radial Basis Function (RBF) interpolation in ArcGIS (Figure 2). The legend in Figure 2 displays the interpolated value ranges of the Formicidae distribution density, with each interval representing a classified density range (e.g., [0, 3.851]). The interpolation results revealed a clustered distribution pattern, with the highest concentrations occurring in the northwestern and northeastern corners, as well as the southeastern region, of the study area. Notably, sample point d2 exhibited the maximum density value, while the western and northern areas showed relatively sparse distributions. Spatial autocorrelation analysis was conducted using Formicidae distribution as the analytical unit. The global Moran’s I index yielded a value of 0.332 (*p* < 0.01; z-score = 119.275), indicating significant positive spatial autocorrelation. These results demonstrate that the Formicidae distribution in the Jinfoping watershed exhibits strong spatial regularity (*p* < 0.01 significance level), pronounced clustering characteristics (Moran’s I > 0.332), and highly non-random distribution patterns (z-score > 2.58).

### 3.2. Model Selection of Spatial Distribution Influence Mechanism

Using the ArcGIS platform, we conducted spatial regression analysis with the spatial distribution of Formicidae abundance as the dependent variable. The independent variables included 18 soil physicochemical parameters (e.g., SOC, BD, and NWC) and seven topographic factors (e.g., TR and EVA). Initial ordinary least squares (OLS) regression analysis revealed multicollinearity issues, with variance inflation factors (VIFs) exceeding 7.5 for several explanatory variables. Following the removal of variables with VIFs > 7.5 to ensure model robustness, we performed a final OLS regression analysis. The complete results of this refined analysis are presented in Table 4.

The spatial autocorrelation analysis revealed significant spatial autocorrelation in Formicidae distribution (*p* < 0.01), indicating non-random clustering patterns. While the ordinary least squares (OLS) model can identify global-scale influencing factors, it fails to capture spatial heterogeneity characteristics. In contrast, the geographically weighted regression (GWR) model adopted in this study effectively revealed local characteristics and spatial differentiation patterns of Formicidae distribution. After eliminating multicollinear variables, the GWR analysis demonstrated superior performance compared to the OLS model. Specifically, the GWR model showed significantly improved goodness-of-fit metrics, including higher R^2^ and adjusted R^2^ values, along with a lower AICc. These results clearly indicate that the GWR model provides a more robust framework for explaining the spatial distribution mechanisms of Formicidae.

### 3.3. Analysis of Influencing Factors on the Spatial Distribution of Formicidae

Within the ArcGIS platform framework, this study utilized the natural breaks classification method to spatially visualize the regression coefficients derived from the geographically weighted regression (GWR) model. This approach effectively reveals the localized effect intensity and spatial heterogeneity of each explanatory variable. Based on their inherent characteristics, the explanatory variables were systematically classified into two distinct categories—soil physicochemical properties and topographic factors—to facilitate a comprehensive analysis.

**Figure 3:**
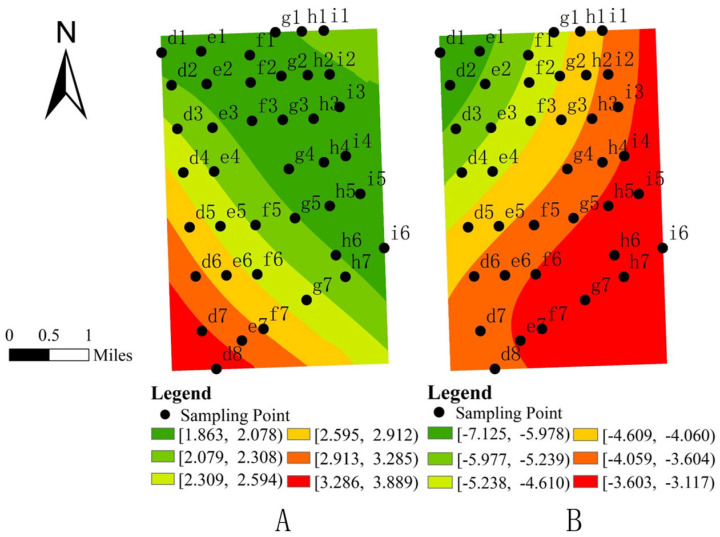
(**A**): Spatial distribution map of available phosphorus (AP) regression coefficients and Formicidae abundance. (**B**): Spatial distribution map of hydrogen peroxidase (HP) regression coefficients and Formicidae abundance.

Our analysis revealed significant spatial relationships between Formicidae abundance and key soil properties, particularly available phosphorus (AP) and hydrogen peroxidase (HP) (Figure 3). AP demonstrated a significant positive correlation with Formicidae abundance, with the strongest effects concentrated in the southwestern portion of the study area and diminishing gradually toward the northeast. In contrast, HP showed a significant negative correlation, exhibiting maximal influence in the northwestern region that progressively weakened in eastern and southern directions.

**Figure 4:**
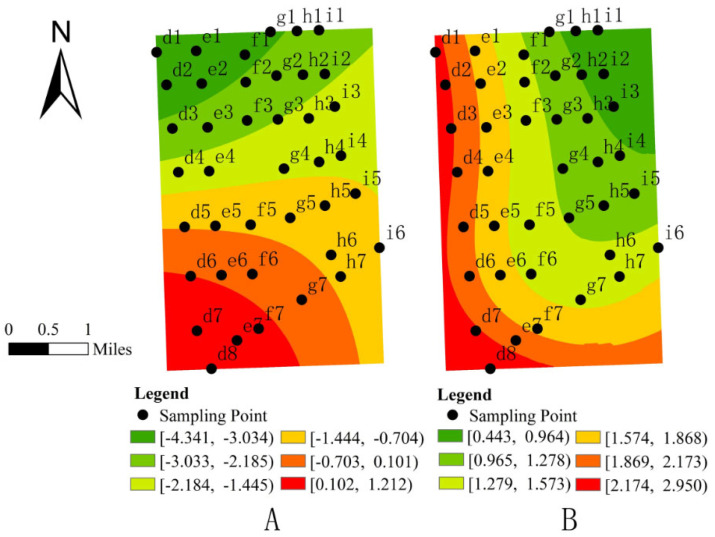
(**A**): Spatial distribution map of regression coefficients for topographic relief (TR) and Formicidae abundance. (**B**): Spatial distribution map of slope (SLP) regression coefficients and Formicidae abundance.

The analysis reveals that topographic factors, particularly topographic relief (TR) and slope (SLP), significantly influence the spatial distribution of Formicidae abundance. Figure 4 demonstrates pronounced spatial heterogeneity in the relationship between TR and Formicidae density. While a general negative correlation is observed across most study areas, a distinct positive correlation emerges in the southwestern region. The influence intensity of TR peaks in the northwestern sector and progressively diminishes along a southwest gradient. Similarly, SLP exhibits a significant positive correlation with Formicidae abundance throughout the study area. The spatial pattern of this relationship shows maximum influence intensity concentrated in the western and southwestern zones, with a gradual weakening effect toward the northeast. These spatially varying relationships highlight the complex interplay between terrain characteristics and Formicidae distribution patterns.

## 4. Discussion

### Analysis of the Spatial Distribution and Influencing Factors of Formicidae

The analysis of spatial distribution patterns using Radial Basis Function (RBF) interpolation revealed the significant clustering of Formicidae populations in the following three distinct regions: the northwestern, northeastern, and southeastern portions of the study area. This aggregated distribution pattern is consistent with the established ecological principles of insect spatial organization observed across multiple ecosystems. Supporting this finding, Catarineu et al. [39] documented similar aggregation behavior in Formicidae colonies within disturbed soil environments in Spain, attributing this pattern to the preferential selection of resource-rich microhabitats. The ant fauna in this study was collected in the litter layer and soil, mostly distributed beneath the deciduous layer. The coverage thickness and decomposition degree of the deciduous layer have been confirmed to be the key microhabitat characteristics affecting the distribution of ant colonies [40,41]. The observed aggregation of Formicidae in this study indicates that these areas provide optimal ecological conditions, including suitable temperature–humidity regimes, abundant food resources, and favorable nesting habitats, collectively promoting their localized clustering.

The global spatial autocorrelation analysis demonstrated significant positive spatial autocorrelation in Formicidae distribution (Moran’s I = 0.332; *p* < 0.01), revealing pronounced clustering patterns within the Jinfoping Small Watershed. These results align with established ecological principles that social insects exhibit non-random spatial organization to optimize colony fitness [13,42,43]. The observed aggregation pattern likely represents an evolutionary adaptation to local environmental conditions, where concentrated populations in resource-rich areas confer multiple ecological advantages. Specifically, this spatial configuration may enhance (1) foraging efficiency through optimized resource exploitation, (2) collective defense against predators and competitors, and (3) social cooperation in brood care and nest maintenance. Such adaptive clustering behavior ultimately contributes to increased survival rates and reproductive success, explaining the persistent spatial patterns observed in the study area.

Model comparison revealed that the geographically weighted regression (GWR) model substantially outperformed the ordinary least squares (OLS) model in terms of goodness-of-fit metrics. This superior performance strongly indicates the presence of significant spatial heterogeneity in Formicidae distribution patterns, demonstrating GWR’s enhanced capability to identify and quantify local-scale ecological drivers [8]. These findings corroborate growing evidence highlighting the critical role of spatial heterogeneity in ecological modeling [44], and reinforce the fundamental importance of incorporating spatial non-stationarity considerations when investigating ecosystem dynamics and species distribution patterns.

The spatial visualization of the GWR model further revealed distinct local-scale relationships between environmental factors and Formicidae distribution patterns [45]. Soil analysis identified available phosphorus (AP) and hydrogen peroxidase (HP) as the most influential physicochemical properties. AP demonstrated a strong positive association with Formicidae abundance. Phosphorus, as an important limiting nutrient in the soil, directly affects the soil microbial activity and plant growth, and thus influences the distribution of Formicidae through the food chain [46]. The results of this study are consistent with those of Frouz et al., showing that ant nests significantly enrich phosphorus through food storage, excreta deposition, and deep soil translocation, resulting in higher available phosphorus (AP) content in nests compared to surrounding soils [47]. In contrast, HP activity showed a significant negative correlation. As a key enzyme in soil redox processes [48], hydrogen peroxidase (HP) may indirectly suppress ant activities through its elevated activity, which alters the soil oxidative environment. Similar mechanisms have been reported in studies of subalpine forests: after naphthalene treatment reduced soil fauna, the activity of polyphenol oxidase (PPO) was significantly decreased, while the activity of peroxidase (POD) was positively correlated with microbial phospholipid fatty acids (PLFAs) [49]. This suggests that the absence of soil fauna may affect soil processes mediated by oxidative enzymes through changes in microbial communities.

Topographic analysis similarly revealed pronounced spatial effects on Formicidae distribution. The slope gradient (SLP) exhibited a positive correlation with ant abundance, with moderate slopes (5–15°) appearing particularly favorable [50], potentially due to enhanced microhabitat diversity and improved drainage conditions that support colony establishment [51]. Conversely, topographic relief (TR) displayed an inverse relationship; the increased topographic relief may impose physical constraints on colony expansion and resource acquisition [52], which aligns with the findings from arid regions [53] and disturbed ecosystems [39].

## 5. Conclusions

(1) The spatial distribution of Formicidae displayed significant clustering patterns, with higher densities predominantly observed in the northwestern and northeastern corners, as well as the southeastern region of the watershed.
(2) Spatial dependence exerted a strong influence on the distribution patterns. Notably, the geographically weighted regression (GWR) model demonstrated a substantially better fit than the ordinary least squares (OLS) model, indicating pronounced spatial heterogeneity in Formicidae distribution.
(3) Spatial visualization analysis further revealed localized effects of soil physicochemical properties and topographic factors. Formicidae abundance exhibited a significant positive correlation with available phosphorus (AP) and slope (SLP), while hydrogen peroxidase (HP) and topographic relief (TR) showed a significant negative correlation.

## Figures and Tables

**Table 1:** Methods of soil physicochemical indices.

Type	Index	Determination Method	Numerical Value
Soil physicochemical index	Natural water content (NWC)	Oven-drying method	6.44 ± 2.71
	Bulk density (BD)	Core sampling method	1.17 ± 0.06
	Saturated water content (SWC)		34.68 ± 5.88
	Non-capillary porosity (NCP)		9.79 ± 2.09
	Total capillary porosity (TCP)		40.2 ± 6.38
	Capillary porosity (CP)		32.1 ± 5.49
	Capillary water holding capacity (CWHC)		27.63 ± 4.71
	Soil organic carbon (SOC)	Dichromate oxidation method	5.65 ± 1.89
	Available potassium (AK)	Flame photometry	73.35 ± 28.56
	Available nitrogen (AN)	Alkaline hydrolysis diffusion method	21.85 ± 13.24
	Available phosphorus (AP)	Molybdenum–antimony spectrophotometric method	6.89 ± 3.91
	Total nitrogen (TN)	Sulfuric acid digestion–sodium salicylate method	0.4 ± 0.15
	Total phosphorus (TP)	Sulfuric acid digestion–molybdenum antimony spectrophotometric method	0.29 ± 0.18
	Potential of hydrogen (pH)	Soil-to-water ratio of 2.5:1	7.93 ± 0.15
	Electrical conductivity (EC)	Determined using a DDS-608 multi-parameter conductivity meter	97.35 ± 12.04
	Hydrogen peroxidase (HP)	Potassium permanganate titration method	2.35 ± 0.78
	Alkaline phosphatase (ALP)	Disodium phenyl phosphate colorimetric method	3.18 ± 0.64
	Urea enzyme (UE)	Starch–phenol blue colorimetric method	3.85 ± 7.1

**Table 2:** Sources of topographic factors.

Type	Index	Source	Numerical Value
Topographic factor	Evaluation (EVA)	NASA Earth Science data website (https://nasadaacs.eos.nasa.gov/) (accessed on 13 December 2024)	18.74 ± 8.04
	Slope (SLP)		179.57 ± 93.57
	Slope variance (SV)		37 ± 19.07
	Slope factor (SF)		82.26 ± 22.7
	Topographic relief (TR)		1377.59 ± 65.51
	Surface roughness (SR)		1.07 ± 0.06
	Aspect (ASP)		12.42 ± 6.25

**Table 3:** Confidence level of the z-score and *p*-value [24].

z-Score (Standard Deviations)	*p*-Value (Probability)	Confidence Level
<−1.65 or >+1.65	<0.10	90%
<−1.96 or >+1.96	<0.05	95%
<−2.58 or >+2.58	<0.01	99%

**Table 4:** Estimation and diagnostic results of the OLS model.

Explanatory Variable	Coefficient	Standard Deviation	T-Value	*p*-Value	VIF
AP	2.832	1.307	2.166	0.037 *	1.062
HP	−4.328	1.344	−3.221	0.003 *	1.122
TR	−1.884	1.338	−1.408	0.168	1.113
SLP	1.553	1.422	1.093	0.282	1.256

Note: * The asterisk next to the number indicates a statistically significant *p*-value (*p* < 0.05).

## Data Availability

The original contributions presented in this study are included in the article. Further inquiries can be directed to the corresponding authors.
